# CFTR Functional Rescue and Heterogeneous Clinical Outcomes Following Elexacaftor/Tezacaftor/Ivacaftor Therapy in People with Cystic Fibrosis Carrying the W1282R Variant

**DOI:** 10.3390/ijms27198896

**Published:** 2026-10-07

**Authors:** Elena Kondratyeva, Anna Efremova, Yuliya Melyanovskaya, Elena Zhekaite, Maria Sapelnikova, Michael Milovanov, Olga Shchagina, Anna Voronkova, Victoria Sherman, Stanislav Krasovsky, Sergey Kutsev, Dmitry Goldshtein

**Affiliations:** 1 Research Centre for Medical Genetics,1 Moskvorechye St., 115552 Moscow, Russia; elenafpk@mail.ru (E.K.); anna.efremova.83@gmail.com (A.E.); elena_zhekayte@mail.ru (E.Z.); krasnova.m.g.0605@gmail.com (M.S.); a199299@ya.ru (M.M.); schagina@med-gen.ru (O.S.); voronkova111@yandex.ru (A.V.); sa_krasovsky@mail.ru (S.K.); mgnc@med-gen.ru (S.K.); dvgoldrm7@gmail.com (D.G.); 2 Independent Researcher, Moscow 121108, Russia

**Keywords:** cystic fibrosis, CFTR, W1282R, elexacaftor/tezacaftor/ivacaftor, CFTR modulators, intestinal current measurement, intestinal organoids, forskolin-induced swelling

## Abstract

The clinical response to elexacaftor/tezacaftor/ivacaftor (ETI) in people with cystic fibrosis carrying the rare cystic fibrosis transmembrane conductance regulator (CFTR) variant W1282R has not been well characterized. We retrospectively analyzed 12 people with cystic fibrosis treated with ETI for 3–36 months in addition to ongoing standard CF therapy. Baseline lung function was generally preserved in patients with available spirometry (median percent predicted forced expiratory volume in 1 s (ppFEV_1_), 110%; interquartile range (IQR), 93–123), limiting the potential for further improvement in ppFEV_1_; no statistically significant change in ppFEV_1_ was detected during ETI therapy (*p* = 0.328). Paired data for ppFEV_1_, body mass index (BMI), and sweat conductivity were available for 8, 12, and 9 patients, respectively. CFTR function was assessed by intestinal current measurement (ICM; *n* = 4) and patient-derived intestinal organoids (*n* = 2). Functional testing demonstrated markedly reduced baseline CFTR activity. In the patient with paired ICM measurements before and during ETI therapy, restoration of CFTR-mediated chloride transport was observed, whereas ETI significantly increased forskolin-induced swelling (FIS) in both patient-derived organoid cultures (*p* < 0.001 for each culture). BMI did not change significantly during ETI therapy (*p* = 0.269). Sweat conductivity showed a statistically significant overall reduction (median change, −3 mmol/L; *p* = 0.031); however, marked reductions were observed in only two of nine patients. Interpretation is limited by the small retrospective cohort and incomplete availability of paired clinical and functional data. These findings indicate that functional responsiveness of W1282R-CFTR to ETI may not be accompanied by consistent improvement across the clinical outcomes assessed.

## 1. Introduction

Cystic fibrosis (CF) is a severe autosomal recessive multisystem disorder caused by pathogenic variants in the gene, encoding the cystic fibrosis transmembrane conductance regulator (CFTR), an adenosine triphosphate (ATP)-binding chloride and bicarbonate channel expressed in epithelial cells. Impaired CFTR function leads to airway surface dehydration, defective mucociliary clearance, chronic inflammation, and progressive lung disease [1].

CFTR modulators have transformed the treatment of CF by targeting the underlying molecular defect. The most pronounced clinical benefits have been achieved with the highly effective triple combination elexacaftor/tezacaftor/ivacaftor (ETI), which improves lung function, nutritional status, and quality of life and reduces the frequency of pulmonary exacerbations in eligible patients [2,3].

Although ETI has substantially improved clinical outcomes in people with cystic fibrosis carrying common ETI-responsive CFTR variants, evidence regarding its effectiveness in rare CFTR variants remains limited. Many rare genotypes were not represented in pivotal clinical trials, making the accumulation of clinical experience and functional data essential for the development of personalized treatment strategies in CF [4,5].

In recent years, functional assays, including intestinal current measurement (ICM) and forskolin-induced swelling (FIS) of patient-derived intestinal organoids, have become important tools for assessing residual CFTR activity and responsiveness of rare CFTR variants to CFTR modulators [4,6]. At the same time, increasing attention has been paid to the potential discrepancy between patient-derived functional responses and clinical outcomes, as restoration of CFTR function in organoid or electrophysiological assays may not always translate into comparable clinical improvement [6,7].

The p.Trp1282Arg (W1282R) variant is a rare missense CFTR variant located in the second nucleotide-binding domain (NBD2), which plays a key role in channel gating and interdomain interactions within CFTR. Unlike the nonsense variant W1282X, which leads to premature translation termination and markedly reduced protein synthesis, W1282R is expected to allow production of full-length CFTR protein. This variant was first described by Ivaschenko et al. in patients with CF from Russia [8]. Functional studies indicate that W1282R is associated with minimal residual CFTR activity. In vitro and ex vivo data nevertheless suggest that W1282R-CFTR retains partial responsiveness to CFTR potentiators and corrector–potentiator combinations [9,10,11].

According to the Russian CF Patient Registry, W1282R accounts for 0.63% of CFTR alleles in Russian patients with CF [12]. Previous studies in Russian cohorts have associated W1282R with a severe CF phenotype and impaired lung function [13]. Subsequent functional studies using intestinal organoids and electrophysiological methods provided preliminary evidence that W1282R may retain responsiveness to CFTR modulators [11].

W1282R is currently included among CFTR variants associated with eligibility for ETI therapy. According to the TRIKAFTA prescribing information, W1282R is listed as responsive to ETI based on in vitro data from the Fisher rat thyroid (FRT) cell assay [14]. However, clinical data on ETI therapy in people carrying W1282R, and particularly data linking ex vivo functional responses with clinical outcomes, remain very limited. To our knowledge, no previous study has combined longitudinal clinical outcomes during ETI therapy in people with CF carrying W1282R with functional data obtained from patient-derived biological material.

The aim of this study was to characterize real-world clinical outcomes during ETI therapy in people with cystic fibrosis carrying the rare CFTR W1282R variant and to examine these outcomes in the context of patient-derived functional assessments of CFTR activity.

## 2. Results

### 2.1. Baseline Patient Characteristics

Clinical data from 12 people with cystic fibrosis (pwCF) carrying the W1282R variant and treated with ETI were retrospectively analyzed. At ETI initiation, patient age ranged from 2 to 32 years, with a median age of 9.8 years (interquartile range (IQR), 5.6–16.0); the cohort included nine children and three adults.

The most common variant in trans with W1282R was CFTRdele2,3, identified in five of 12 patients. The 2143delT variant was identified in two unrelated patients; all other variants were each observed in a single patient.

Baseline lung function was generally preserved in patients with available spirometry. Most children and adolescents had percent predicted forced expiratory volume in 1 s (ppFEV_1_) values exceeding 100% predicted. Among the three adult patients, two had relatively preserved lung function, whereas one had severe lung function impairment (ppFEV_1_ < 40% predicted).

Intermittent or chronic *Pseudomonas aeruginosa* infection was documented in 50.0% of patients, and chronic rhinosinusitis with nasal polyps was present in 75.0%. All patients had pancreatic insufficiency.

The main demographic and baseline clinical characteristics of the study population are summarized in Table 1, with individual patient characteristics presented in Table 2.

ETI was initiated in addition to ongoing standard CF therapy. Background CF therapy at ETI initiation and during follow-up is summarized in Table 3. No major treatment changes were documented.

### 2.2. Clinical Outcomes During ETI Therapy

Twelve patients received ETI for 3–36 months. Clinical outcomes were analyzed using paired measurements obtained before and during ETI therapy (Table 4).

Paired ppFEV_1_ data were available for eight patients. There was no statistically significant difference between baseline and follow-up values (*p* = 0.328).

Paired body mass index (BMI) data were available for 12 patients. The median change in BMI was −0.8 kg/m^2^ (range, −4.7 to +3.4; *p* = 0.269). BMI changes were small or negative in most patients. The greatest increases were observed in P6 (+2.4 kg/m^2^) and P7 (+3.4 kg/m^2^). In P7, the increase in BMI was accompanied by a 39 mmol/L decrease in sweat conductivity, which reached the intermediate range.

Paired sweat conductivity measurements during ETI therapy were available for nine patients. The median change in sweat conductivity was −3 mmol/L (range, −59 to +1; *p* = 0.031). In most patients, sweat conductivity remained within the abnormal range despite ETI therapy. The greatest reduction was observed in P3, in whom sweat conductivity decreased from 100 to 41 mmol/L, reaching the normal range. Apart from the marked reductions observed in P3 and P7, individual changes were small, ranging from −8 to +1 mmol/L.

Follow-up data on sinonasal disease were available for six patients. Despite ETI therapy, progression of chronic rhinosinusitis with nasal polyps was observed in several patients. P2 and P6 developed progressive polyposis with complete opacification of the paranasal sinuses. P3 required surgical treatment for chronic rhinosinusitis with nasal polyps (CRSwNP) two years after ETI initiation. P4, who had undergone sinus surgery before starting ETI, required repeat surgery after three years of treatment. No improvement in sinonasal disease was observed in P9 or P11.

Overall, the clinical response to ETI in patients carrying W1282R was heterogeneous, with variable changes in respiratory, nutritional, and CFTR-related biomarkers. Although marked reductions in sweat conductivity and improvements in nutritional status were observed in individual patients, consistent improvement in lung function was not observed across the cohort, and sinonasal disease continued to progress in several patients.

**Figure 1:**
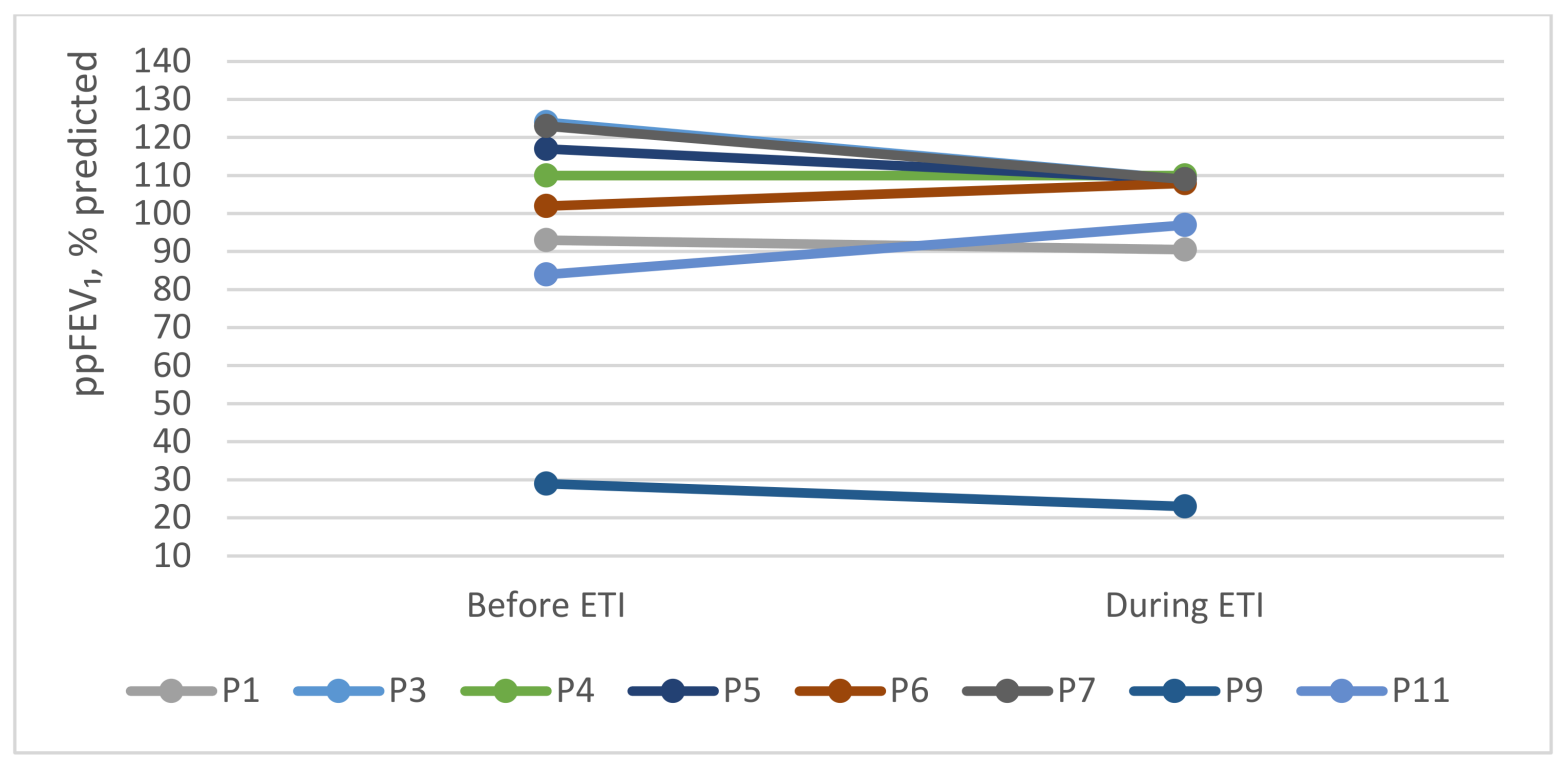
Changes in ppFEV_1_ during ETI therapy in patients with paired measurements (*n* = 8 of 12).

**Figure 2:**
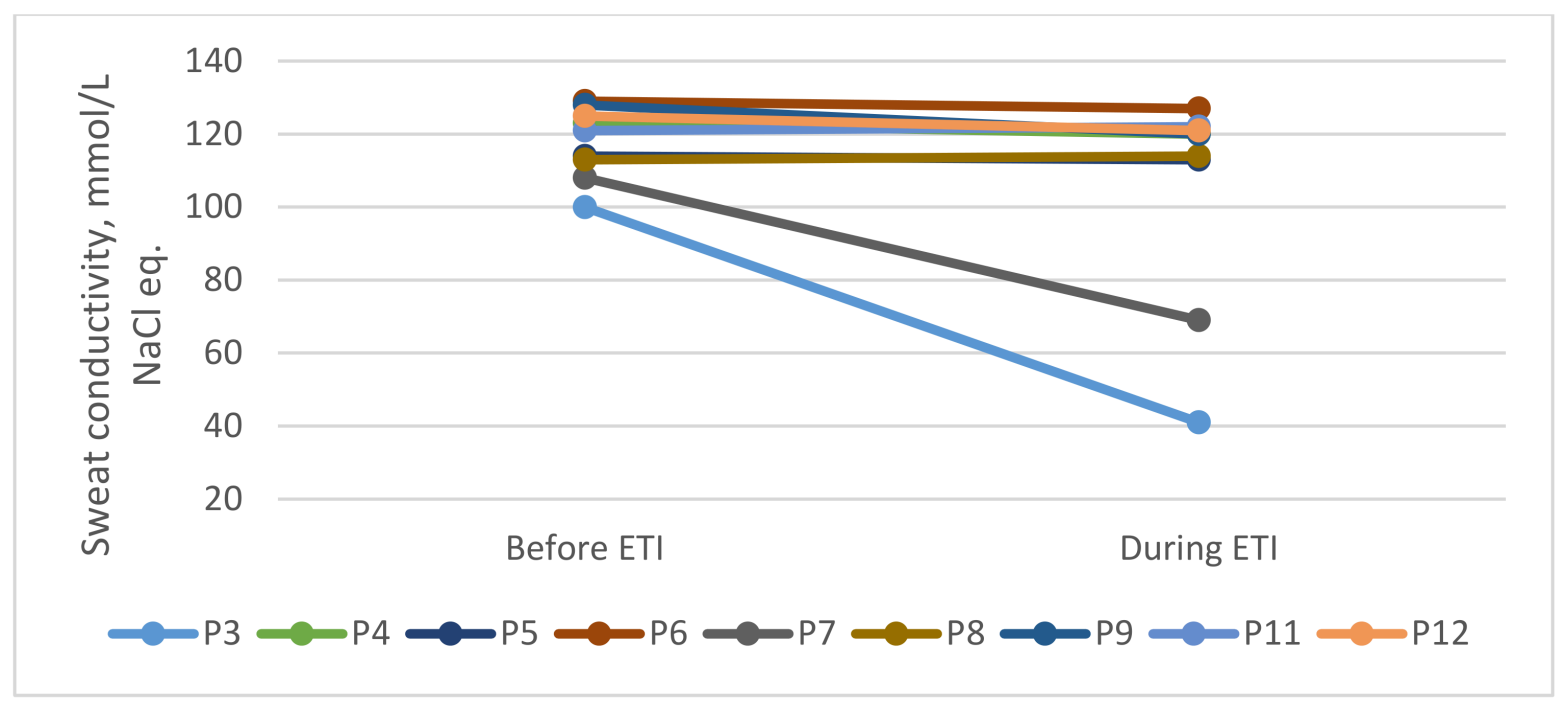
Changes in sweat conductivity during ETI therapy in patients with paired measurements (*n* = 9 of 12).

**Figure 3:**
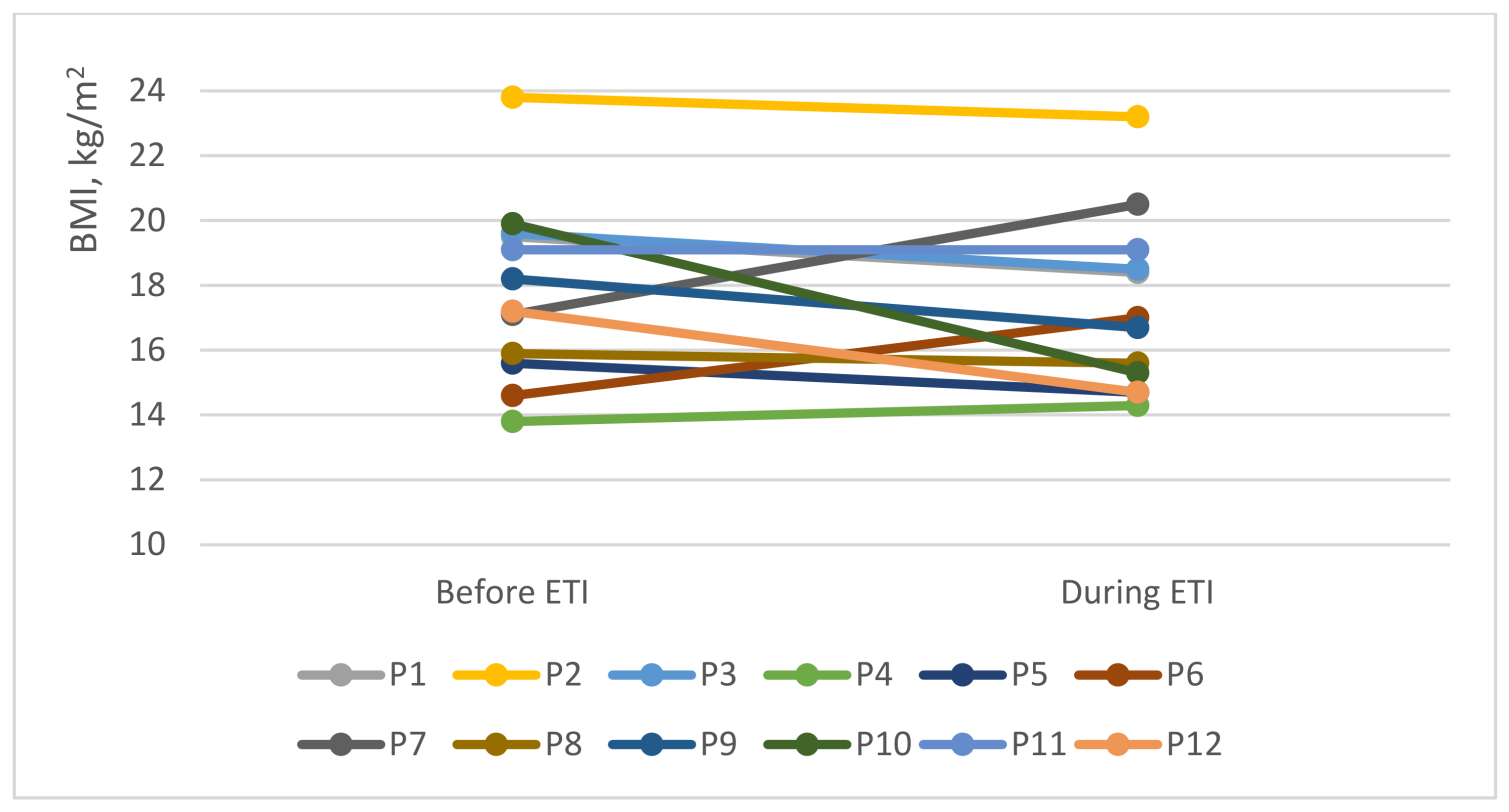
Changes in BMI during ETI therapy in patients with paired measurements (*n* = 12).

Changes in ppFEV_1_, sweat conductivity, and BMI in patients with paired measurements are shown in Figure 1, Figure 2 and Figure 3.

### 2.3. Functional Assessment of Response to ETI

CFTR function was assessed in patients carrying the W1282R variant using two independent functional assays: intestinal current measurement (ICM) and the forskolin-induced swelling (FIS) assay in patient-derived intestinal organoids.

Pre-ETI ICM data were available for two patients with the W1282R/CFTRdele2,3 genotype (P5 and P6). In both patients, the forskolin-induced response was minimal (2.17 ± 0.54 and 1.17 ± 0.41 μA/cm^2^, respectively; Table 5), indicating markedly impaired CFTR-mediated chloride transport. These values were comparable to those observed in the F508del/F508del reference group.

**Figure 4:**
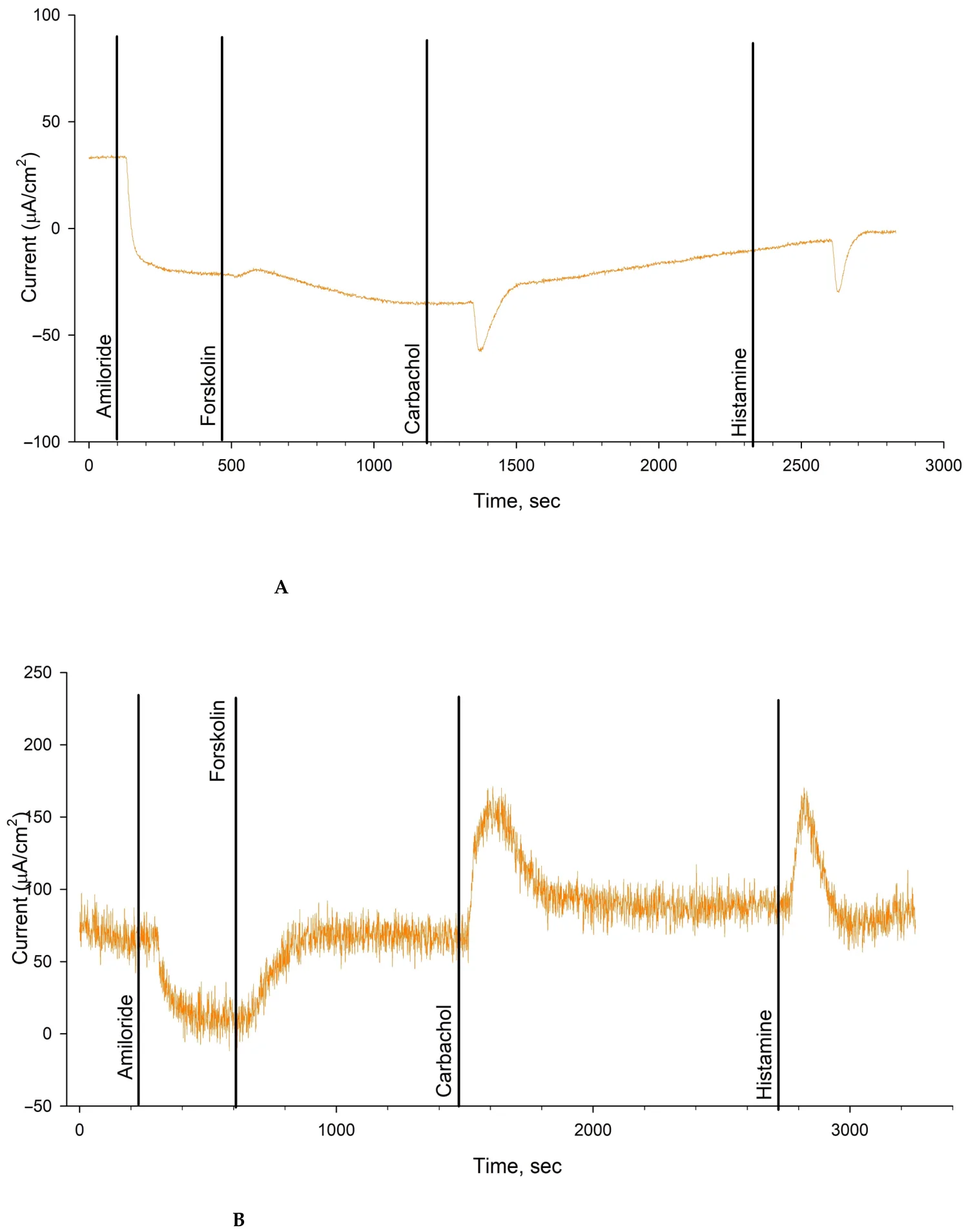
Intestinal current measurement (ICM) in patient P6 before and during ETI therapy. Representative ICM tracings obtained before ETI initiation (**A**) and after two years of ETI therapy (**B**) showing responses to sequential stimulation with amiloride, forskolin, carbachol, and histamine.

In P6, repeat ICM after two years of ETI therapy showed a marked increase in forskolin-induced current from 1.17 ± 0.41 to 24.17 ± 6.02 μA/cm^2^ (Figure 4), indicating a substantial increase in CFTR-mediated chloride transport. In P4 (W1282R/CFTRdele2,3) and P8 (W1282R/394delTT), ICM was performed only during ETI therapy. Both patients showed marked forskolin-induced responses (13.33 ± 2.30 and 20.17 ± 0.29 μA/cm^2^, respectively), comparable to the response observed in P6 during ETI therapy and approaching the values observed in healthy controls.

Data are presented as mean ± standard deviation (SD). Reference values for the F508del/F508del group and healthy controls were obtained from Melyanovskaya et al. [15,16].

Additional functional assessment was performed in P4 and P8 using patient-derived intestinal organoids. Organoids with the F508del/F508del genotype, a well-characterized reference genotype in intestinal organoid-based CFTR functional studies, were used as a reference culture [17,18,19].

**Figure 5:**
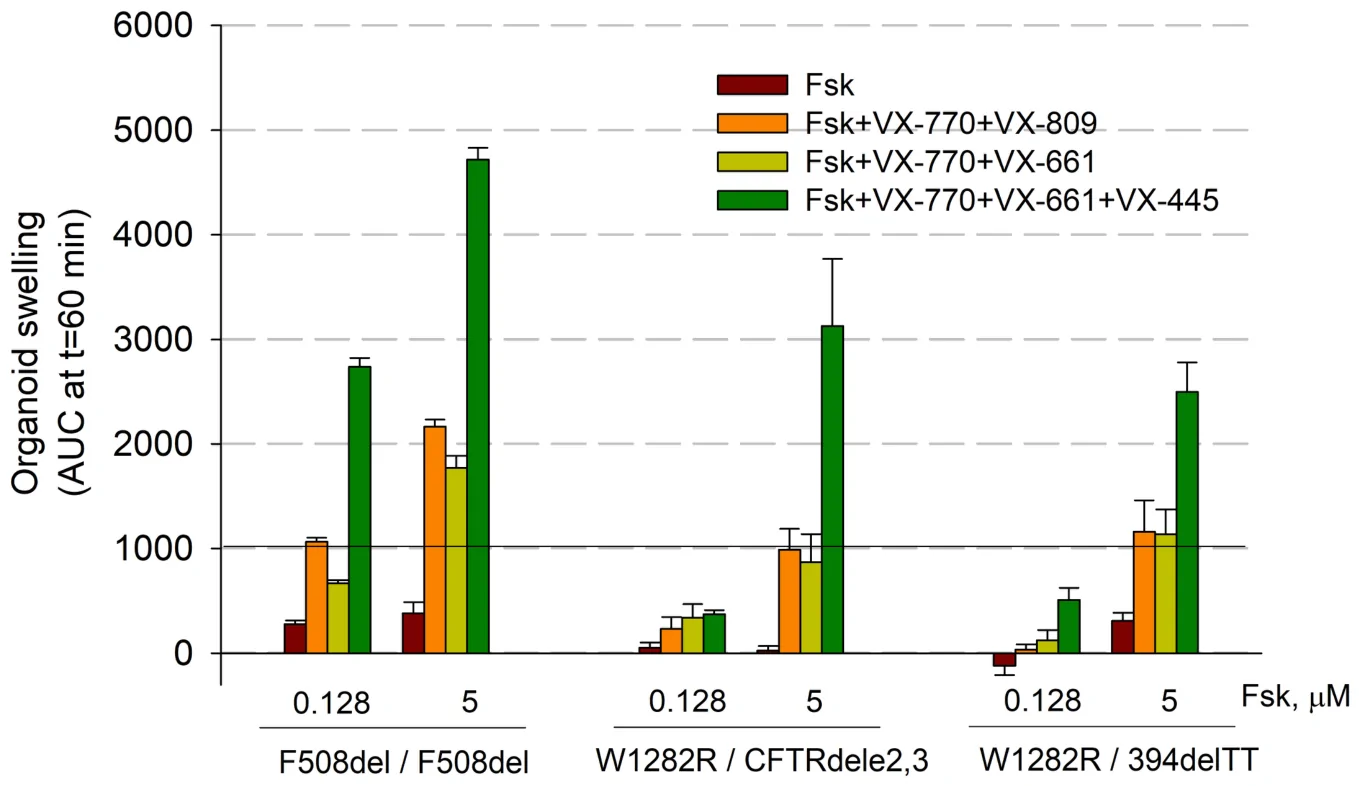
Quantitative assessment of forskolin-induced swelling in intestinal organoids carrying the W1282R variant at 0.128 and 5 μM forskolin. F508del/F508del organoids were used as a reference culture. VX-770 + VX-809, ivacaftor/lumacaftor; VX-770 + VX-661, ivacaftor/tezacaftor; VX-770 + VX-661 + VX-445, ivacaftor/tezacaftor/elexacaftor. For each W1282R organoid culture, *n* = 1 biological replicate with 12 technical replicates. For the F508del/F508del reference culture, *n* = 3 biological replicates with 12 technical replicates per culture.

**Figure 6:**
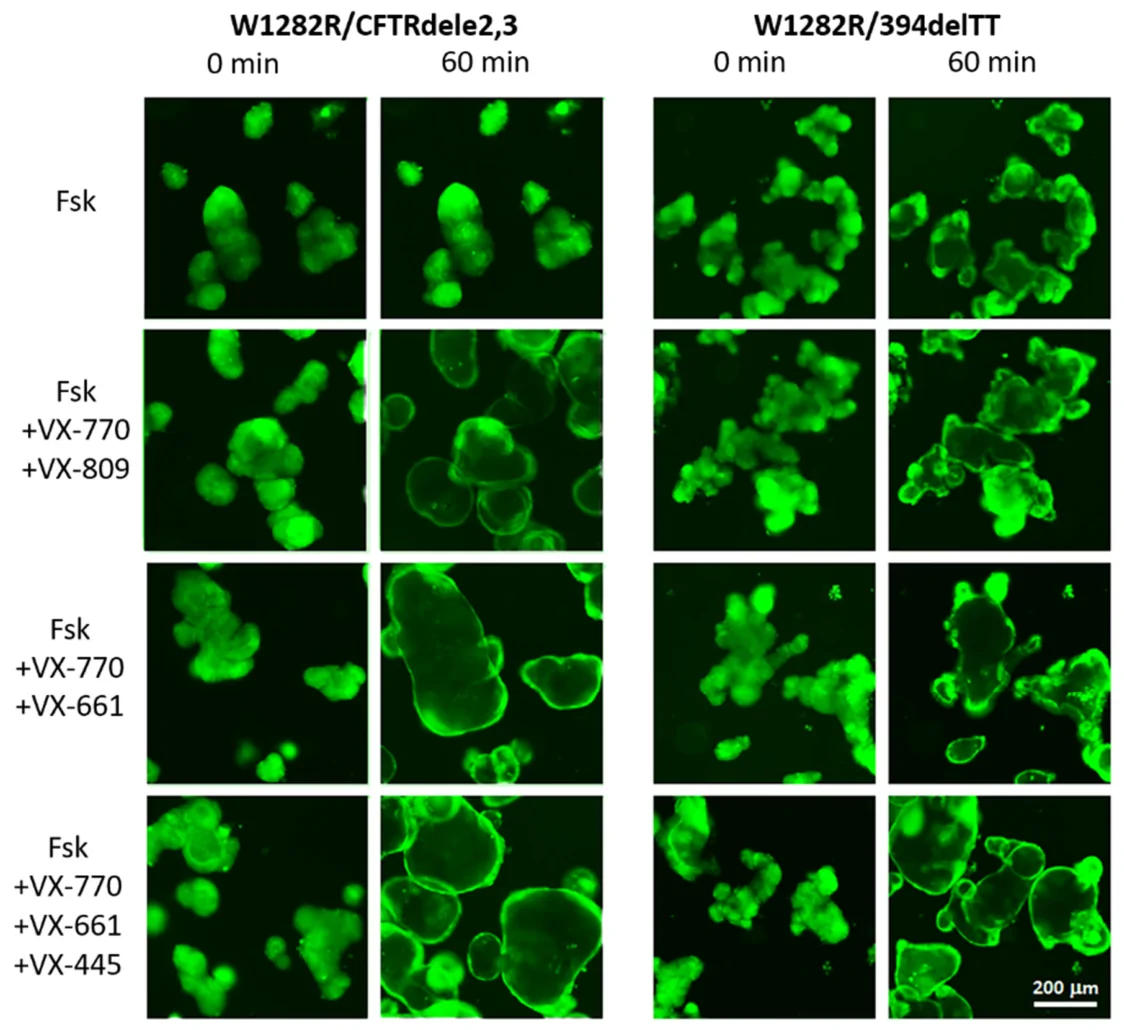
Representative fluorescence micrographs of intestinal organoids before and after forskolin stimulation in the presence of CFTR modulators. Organoids were stimulated with 5 μM forskolin; each CFTR modulator was used at a concentration of 3.5 μM. Organoids were stained with Calcein (0.84 μM, 30 min). Scale bar, 200 μm.

In the absence of CFTR modulators, W1282R organoids showed very low residual CFTR activity. Following stimulation with 5 μM forskolin, W1282R/394delTT (P8) organoids exhibited minimal swelling, similar to that observed in F508del/F508del organoids, whereas W1282R/CFTRdele2,3 (P4) organoids showed virtually no response to forskolin stimulation (Figure 5 and Figure 6).

Incubation with CFTR modulator combinations resulted in forskolin-induced organoid swelling, indicating increased CFTR-mediated fluid transport. The greatest FIS response was observed with elexacaftor/tezacaftor/ivacaftor. Compared with forskolin alone, ETI significantly increased FIS in both W1282R organoid cultures (*p* < 0.001 for each culture). In both W1282R organoid cultures, area under the curve (AUC) values reached approximately 3000 arbitrary units (a.u.), whereas the response in the F508del/F508del reference culture was greater, with an AUC of approximately 4700 a.u.

Taken together, findings from ICM and intestinal organoid assays were consistent with very low baseline CFTR activity associated with the W1282R variant and demonstrated responsiveness to CFTR modulators. The most compelling evidence was obtained in P6, for whom ICM data were available both before and during ETI therapy and showed a marked increase in CFTR-mediated chloride transport. Intestinal organoid studies further demonstrated a functional response of W1282R-CFTR to ETI, although the magnitude of the response remained lower than that observed in the F508del/F508del reference culture.

Despite evidence of increased CFTR function in ICM and intestinal organoid assays, clinical outcomes in the functionally assessed patients were heterogeneous. In P4, a positive functional response was accompanied by stable, high baseline ppFEV_1_ and no meaningful change in sweat conductivity. In P6, the marked increase in CFTR-mediated chloride transport was accompanied by a 6-percentage-point increase in ppFEV_1_ and a 2.4 kg/m^2^ increase in BMI, whereas sweat conductivity remained essentially unchanged. In P8, a functional response was demonstrated by two independent assays, with no improvement in nutritional status or change in sweat conductivity. Thus, evidence of increased CFTR function was not consistently accompanied by marked improvement in conventional clinical outcomes (Table 6).

## 3. Discussion

The main finding of this study is that W1282R-CFTR demonstrated responsiveness to CFTR modulators in patient-derived functional assays, whereas clinical outcomes during ETI therapy were heterogeneous and did not show consistent improvement. However, the limited availability of paired functional data did not allow us to assess the relationship between functional and clinical responses across the whole cohort.

Functional studies showed that the W1282R variant is associated with very low residual CFTR channel activity. In patients with the W1282R/CFTRdele2,3 genotype, minimal forskolin-induced currents were recorded by ICM before treatment, while intestinal organoid testing showed absent or very low swelling in response to forskolin stimulation. Nevertheless, both methods demonstrated responsiveness of the W1282R variant to CFTR modulators. Intestinal organoid testing showed that W1282R responded to both ivacaftor/lumacaftor and ivacaftor/tezacaftor; however, the magnitude of forskolin-induced swelling remained substantially lower than that observed in the F508del/F508del reference culture. Similar results were previously obtained by our group in a patient with the W1282R/CFTRdele2,3 genotype, in whom responsiveness to CFTR modulators was also demonstrated [11].

Carbachol- and histamine-induced responses in ICM cannot be considered exclusively CFTR-mediated, as Ca^2+^-dependent ion transport pathways may also contribute to these responses [20]. Therefore, the relative contribution of CFTR and Ca^2+^-dependent components cannot be determined from these responses alone. In contrast, the marked increase in the forskolin-induced current observed in P6 during ETI therapy provides more direct evidence of increased CFTR-mediated chloride transport.

In the present study, the greatest functional response was observed with elexacaftor/tezacaftor/ivacaftor. Overall, these findings indicate partial rescue of W1282R-CFTR function by CFTR modulators. The clinical consequences of this partial rescue, however, appear to vary considerably between patients.

Interpretation of the clinical response in this study is complicated by the characteristics of the cohort. Unlike clinical trials of CFTR modulators, in which the baseline FEV_1_ range is usually restricted by inclusion criteria, the present study reflects real-world clinical practice after the expansion of ETI indications to rare CFTR variants. Patients were included irrespective of baseline lung function, resulting in heterogeneity of clinical characteristics. Most patients with available spirometry had preserved baseline lung function, which limited the potential for further improvement in ppFEV_1_. Berkers et al. showed that the correlation between intestinal organoid response and change in ppFEV_1_ was weaker in patients with baseline ppFEV_1_ < 40% or >90%, consistent with irreversible structural lung damage and a ceiling effect, respectively, whereas the association with change in sweat chloride concentration was preserved [6]. Therefore, the absence of a marked increase in lung function in some patients in our cohort does not exclude CFTR functional rescue during ETI therapy and should be interpreted in light of baseline lung function and the extent of structural lung disease. These factors are particularly relevant to the interpretation of ppFEV_1_ and other downstream clinical outcomes but are less likely to account for variability in the sweat test, which more directly reflects CFTR-dependent ion transport.

Heterogeneity of response was also observed in sweat test results. Although the median change in sweat conductivity in the cohort was only −3 mmol/L, individual responses varied considerably: in some patients, sweat conductivity decreased by 39–59 mmol/L, and in one patient it reached the normal range. At the same time, sweat conductivity remained above the normal range in most patients. These findings indicate marked interindividual variability in the in vivo response to ETI. Importantly, in several functionally assessed patients, clear evidence of CFTR responsiveness in intestinal assays was accompanied by little or no change in the sweat test. This discordance cannot readily be explained by baseline lung function or structural lung disease and suggests that additional factors may contribute to differences in treatment response across tissues.

Recent data on the relationship between the degree of CFTR functional restoration and the clinical effect of ETI provide additional context for these findings. In a recent real-world study, Burgel et al. showed that patients with two ETI-responsive CFTR variants had a greater reduction in sweat chloride concentration than those with only one responsive variant, whereas the magnitude of ppFEV_1_ improvement did not differ between the groups. The authors suggested that irreversible lung damage, rather than insufficient correction of CFTR function, might limit the achievement of normal lung function [21]. These observations support the distinction between restoration of CFTR function and improvement in downstream clinical outcomes such as lung function.

The course of chronic rhinosinusitis with nasal polyps provides additional evidence of heterogeneous clinical response. Despite evidence of CFTR responsiveness in patient-derived assays, sinonasal polyposis persisted or progressed in some patients, and surgical treatment was required in individual cases, further suggesting that treatment response may differ between affected organs.

Patient-derived intestinal functional assays remain clinically relevant. Previous studies have demonstrated associations between CFTR modulator responses in intestinal organoids and in vivo therapeutic outcomes, including changes in ppFEV_1_ and sweat chloride [6,22,23,24]. However, these associations do not imply identical correction across tissues or precise prediction of clinical response in an individual patient. Tissue-specific differences in CFTR expression and modulator responsiveness, including differences between intestinal and respiratory epithelia, may therefore contribute to discordant responses across outcome measures.

Tissue-dependent differences in response to CFTR modulators have also been described for the N1303K variant. According to Pranke et al., ETI therapy was associated with restoration of CFTR function in functional assays, while sweat test values remained abnormal in some patients. The authors interpreted this as a manifestation of tissue-specific and incomplete restoration of N1303K-CFTR function [7]. Our findings suggest that a similar response pattern may also occur in patients carrying the W1282R variant.

The practical implication of our findings is that positive ICM and intestinal organoid results demonstrate responsiveness to CFTR modulators but should not be regarded as direct predictors of the magnitude of clinical response in an individual patient. These findings indicate that the inclusion of W1282R in the list of ETI-responsive variants does not by itself guarantee a clinically meaningful response in every patient and underscore the need for comprehensive assessment of treatment effectiveness, particularly in patients whose second CFTR variant is not expected to result in functional CFTR protein production. The data presented here expand current understanding of the clinical response to ETI in rare CFTR variants and highlight the importance of integrating functional and clinical methods when evaluating treatment effectiveness. Further studies in larger cohorts are needed to clarify the clinical significance of these observations.

The main limitations of this study are its retrospective design, the small cohort inherent to the rarity of the W1282R variant, and incomplete longitudinal clinical and functional data, with variable follow-up duration. Nevertheless, this cohort reflects real-world clinical practice following the expansion of ETI indications to rare CFTR variants. The combination of clinical observations with two independent functional methods provided additional insight into the response pattern of the W1282R variant to ETI therapy and helped define directions for future research.

## 4. Materials and Methods

### 4.1. Study Design and Patients

We retrospectively analyzed clinical and functional data from people with cystic fibrosis (pwCF) carrying the W1282R variant who were treated with elexacaftor/tezacaftor/ivacaftor (ETI). Patients were identified through the Russian Cystic Fibrosis Patient Registry and were followed at regional cystic fibrosis centers across the Russian Federation.

The diagnosis of CF was established according to accepted Russian and international diagnostic criteria [25,26]. The inclusion criterion was the presence of W1282R in compound heterozygosity with another CFTR variant that did not independently confer eligibility for ETI therapy.

The clinical cohort included 12 pwCF carrying the W1282R variant. Functional assessment of CFTR was performed in a subgroup of patients. ICM data were available for four patients: one patient had paired measurements before and during ETI therapy, one was assessed only before ETI initiation, and two were assessed only during treatment. Intestinal organoid testing was performed in two patients.

Clinical data were obtained from medical records and the Russian Cystic Fibrosis Patient Registry. Baseline characteristics before ETI initiation and available follow-up data during treatment were analyzed.

### 4.2. Molecular Genetic Analysis

CFTR genotypes were obtained from medical records and the Russian Cystic Fibrosis Patient Registry. Molecular genetic testing had been performed previously as part of routine diagnostic assessment. The identified CFTR variants were reviewed using international CFTR variant databases.

### 4.3. CFTR Modulator Therapy

Patients received elexacaftor/tezacaftor/ivacaftor (ETI) at age-appropriate doses in accordance with the prescribing information and current clinical guidelines. The duration of follow-up during ETI therapy ranged from 3 to 36 months. The analysis included baseline data obtained before ETI initiation and the latest available follow-up data during treatment.

ETI was administered in addition to standard CF therapy, including mucolytic and antimicrobial therapy and pancreatic enzyme replacement, which was continued as clinically indicated during the follow-up period.

### 4.4. Clinical Assessment

Baseline clinical characteristics included lung function, nutritional status, and airway microbiological status. Clinical outcomes during ETI therapy were assessed based on changes in percent predicted forced expiratory volume in 1 s (ppFEV_1_), body mass index (BMI), and sweat conductivity. Available data on the course of chronic rhinosinusitis with nasal polyps (CRSwNP) during ETI therapy were also reviewed.

Individual changes in ppFEV_1_, BMI, and sweat conductivity were calculated as the difference between the value obtained during ETI therapy and the corresponding baseline value before ETI initiation.

### 4.5. Sweat Conductivity Measurement

Sweat conductivity was measured using the Nanoduct^®^ Neonatal Sweat Analysis System (Model 1030; ELITechGroup Inc., Logan, UT, USA), which is the most commonly used sweat test method in the Russian Federation. Results were expressed as mmol/L NaCl equivalent. Values < 50 mmol/L NaCl equivalent were considered normal, values of 50–79 mmol/L NaCl equivalent intermediate, and values ≥ 80 mmol/L NaCl equivalent abnormal [27]. Paired sweat conductivity measurements obtained before ETI initiation and during treatment were used to assess individual changes during ETI therapy.

### 4.6. Intestinal Current Measurement

Intestinal current measurement (ICM) was performed according to the European Standard Operating Procedure (SOP V2.7, 26 October 2011) as previously described by Derichs et al. [28].

Rectal mucosal biopsies (3–5 mm) were obtained using disposable endoscopic biopsy forceps (Olympus EndoJaw™; Olympus, Tokyo, Japan). ICM findings were interpreted using previously established reference values from healthy controls and pwCF homozygous for F508del [16].

ICM was performed at the Cystic Fibrosis Research and Clinical Department of the Research Centre for Medical Genetics (Moscow, Russian Federation) [29].

Because ICM was available only for a limited number of patients, the results were analyzed individually.

### 4.7. Intestinal Organoid Generation and Culture

Patient-derived intestinal organoids were generated from rectal biopsy specimens. Intestinal crypts were isolated from the biopsy material, embedded in Matrigel, and cultured in standard intestinal organoid growth medium as previously described [30,31].

### 4.8. Forskolin-Induced Swelling Assay

CFTR function in intestinal organoids was assessed using the forskolin-induced swelling (FIS) assay. Forskolin stimulates CFTR-mediated ion and fluid transport into the organoid lumen, resulting in organoid swelling. Changes in organoid area over time were used to assess CFTR channel function.

For the FIS assay, organoids were seeded into 96-well plates, and the CFTR correctors lumacaftor (VX-809), tezacaftor (VX-661), and elexacaftor (VX-445) were added to the corresponding wells at a concentration of 3.5 μM. After 24 h, the organoids were stained with 0.84 μM Calcein AM (BioLegend, San Diego, CA, USA), and the plates were placed in a CO_2_ incubation chamber of a Zeiss Axiovert 5 fluorescence microscope (Carl Zeiss Microscopy, Jena, Germany) equipped with a motorized stage. Images of all samples were acquired at t = 0 min. Forskolin (0.128 or 5 μM) and the CFTR potentiator ivacaftor (VX-770; 3.5 μM) were then added, and the same fields were imaged every 10 min for 60 min.

Quantitative analysis of organoid swelling was performed using custom-developed Analyzer_v5 software. The area under the curve (AUC) for the change in organoid area over time was calculated using Microsoft Excel (2019). Statistical analysis of the experimental data was performed using SigmaPlot 14.5.

For each W1282R genotype, intestinal organoids were available from one patient (*n* = 1 biological replicate). The FIS assay was performed in two independent experimental runs, with six technical replicates per experiment, yielding a total of 12 technical measurements for each W1282R organoid culture. For the F508del/F508del reference genotype, organoids from three patients were analyzed (*n* = 3 biological replicates), with 12 technical replicates per culture. All culture procedures were performed at 37 °C in a humidified atmosphere containing 5% CO_2_.

### 4.9. Analysis of Clinical and Functional Responses

Clinical response was assessed based on changes in ppFEV_1_, BMI, and sweat conductivity. In addition, available medical record data on the course of chronic rhinosinusitis with nasal polyps (CRSwNP), including disease progression and the need for surgical treatment, were reviewed.

Functional response was assessed using ICM and the FIS assay in intestinal organoids.

Given the small sample size and variable data availability, the analysis was performed at two levels: a group-level analysis of the clinical cohort for parameters available in most patients and an individual-level analysis of the functional subgroup comprising patients who underwent ICM and/or intestinal organoid testing.

### 4.10. Statistical Analysis

Given the small sample size and variable availability of paired data, the statistical analysis was primarily descriptive. Quantitative clinical variables were summarized using medians with interquartile ranges (IQRs) or ranges, as appropriate, and categorical variables as absolute numbers and percentages. Individual changes during ETI therapy were calculated as the difference between follow-up and baseline values. Paired clinical measurements obtained before and during ETI therapy were compared using the Wilcoxon signed-rank test. A two-sided *p* value < 0.05 was considered statistically significant.

## Figures and Tables

**Table 1:** Baseline Demographic and Clinical Characteristics of the Study Population.

Characteristic	Value
Patients, *n*	12
Male sex, *n* (%)	4 (33.3)
Age at ETI initiation, years, median (IQR) Children, *n* (%) Adults, *n* (%)	9.8 (5.6–16.0) 9 (75.0) 3 (25.0)
CFTR variant in trans with W1282R CFTRdele2,3, *n* (%) 2143delT, *n* (%) Other variants (R785X, 1898+1G->A, G542X, 394delTT, 3821delT), *n* (%)	5 (41.7) 2 (16.7) 5 (41.7)
Age at diagnosis, years, median (IQR)	1.0 (0.2–1.5)
ppFEV_1_, % predicted, median (IQR)	110 (93–123)
Body mass index (BMI), kg/m^2^, median (IQR)	17.7 (15.8–19.5)
Pancreatic insufficiency, *n* (%)	12 (100)
*Pseudomonas aeruginosa* infection, *n* (%)	6 (50.0)
Chronic rhinosinusitis with nasal polyps (CRSwNP), *n* (%)	9 (75.0)

**ETI, elexacaftor/tezacaftor/ivacaftor; IQR, interquartile range; CFTR, cystic fibrosis transmembrane conductance regulator; ppFEV_1_, percent predicted forced expiratory volume in 1 s.**

**Table 2:** Baseline clinical characteristics of individual patients.

Patient	Genotype	Sex	Age at Diagnosis, Years	Age at ETI Initiation, Years	Baseline ppFEV_1_, % Predicted	Baseline Sweat Conductivity, mmol/L NaCl Equivalent	*P. aeruginosa*
P1	W1282R/R785X	F	3	19	93	NA	−
P2	W1282R/CFTRdele2,3	F	0.3	14	161	95	+
P3	W1282R/CFTRdele2,3	F	1.4	15	124	100	+/−
P4	W1282R/CFTRdele2,3	M	0.2	6	110	123	−
P5	W1282R/CFTRdele2,3	M	0.1	6	117	114	−
P6	W1282R/CFTRdele2,3	M	0.1	6	102	129	−
P7	W1282R/G542X	M	1.7	13.6	123	108	+
P8	W1282R/394delTT	F	0.9	4.5	ND	113	+
P9	W1282R/1898+1G>A	F	3	32	29	128	+
P10	W1282R/2143delT	F	1.4	2	ND	120	+
P11	W1282R/3821delT	F	1.0	22.4	84	121	Bcc
P12	W1282R/2143delT	F	0.1	2.5	ND	125	−

NA, data not available; ND, spirometry not performed because of age; +, chronic *Pseudomonas aeruginosa* infection; +/−, intermittent *P. aeruginosa* infection; −, no *P. aeruginosa* infection; Bcc, Burkholderia cepacia complex. ETI, elexacaftor/tezacaftor/ivacaftor.

**Table 3:** Background cystic fibrosis therapy at ETI initiation and during follow-up.

Patient	Background CF Therapy
P1	Dornase alfa; hypertonic saline; pancreatic enzyme replacement therapy (PERT)
P2	Dornase alfa; hypertonic saline; chronic inhaled antibiotics; PERT; ursodeoxycholic acid; acid-suppressive therapy; intranasal corticosteroid
P3	Dornase alfa; chronic inhaled antibiotics; PERT; ursodeoxycholic acid; intranasal corticosteroid
P4	Dornase alfa; hypertonic saline; PERT; ursodeoxycholic acid; acid-suppressive therapy; intranasal corticosteroid
P5	Dornase alfa; hypertonic saline; PERT; ursodeoxycholic acid; acid-suppressive therapy; intranasal corticosteroid
P6	Dornase alfa; hypertonic saline; PERT; ursodeoxycholic acid; intranasal corticosteroid
P7	Dornase alfa; hypertonic saline; chronic inhaled antibiotics; long-term azithromycin; PERT; ursodeoxycholic acid
P8	Dornase alfa; hypertonic saline; chronic inhaled antibiotics; PERT; ursodeoxycholic acid
P9	Dornase alfa; hypertonic saline; chronic inhaled antibiotics; long-term azithromycin; PERT; salbutamol; intranasal corticosteroid
P10	Dornase alfa; hypertonic saline; chronic inhaled antibiotics; PERT; ursodeoxycholic acid
P11	Dornase alfa; hypertonic saline; chronic inhaled antibiotics; PERT; salbutamol; intranasal corticosteroid
P12	Dornase alfa; hypertonic saline; PERT; ursodeoxycholic acid

**Table 4:** Individual clinical and functional outcomes during ETI therapy.

Patient	Age at ETI Initiation, Years	Follow-Up, Months	ppFEV_1_, % Predicted			BMI, kg/m^2^			Sweat Conductivity, mmol/L NaCl Equivalent			ICM	FIS
			Before ETI	During ETI	∆	Before ETI	During ETI	∆	Before ETI	During ETI	∆		
P1	19	36	93	90.5	−2.5	19.5	18.4	−1.1	NA	NA			
P2	14	10	161	NA		23.8	23.2	−0.6	95	NA			
P3	15	12	124	109	−15	19.6	18.5	−1.1	100	41	−59		
P4	6	12	110	110	0	13.8	14.3	+0.5	123	120	−3	+	+
P5	6	12	117	109	−8	15.6	14.7	−0.9	114	113	−1	+	
P6	6	12	102	108	+6	14.6	17	+2.4	129	127	−2	+	
P7	13.6	12	123	109	−14	17.12	20.55	+3.4	108	69	−39		
P8	4.5	8	ND	ND		15.9	15.6	−0.3	113	114	+1	+	+
P9	32	36	29	23	−6	18.2	16.7	−1.5	128	120	−8		
P10	2	8	ND	ND		19.98	15.3	−4.7	120	NA	NA		
P11	22.4	36	84	97	+13	19.1	19.1	0	121	122	+1		
P12	2.5	3	ND	ND		17.2	14.7	−2.5	125	121	−4		

NA, data not available; ND, spirometry not performed because of age; ppFEV_1_, percent predicted forced expiratory volume in 1 s; BMI, body mass index; ICM, intestinal current measurement; FIS, forskolin-induced swelling; ETI, elexacaftor/tezacaftor/ivacaftor; Δ, change from baseline.

**Table 5:** Changes in short-circuit current density (ΔI_SC_) in response to sequential stimulation during ICM.

ΔI_SC_, µA/cm^2^	Amiloride	Forskolin	Carbachol	Histamine
P5, before ETI (W1282R/CFTRdele2,3)	−18.67 ± 2.94	2.17 ± 0.54	11.33 ± 3.17	11.83 ± 2.35
P6, before ETI (W1282R/CFTRdele2,3)	−24.67 ± 1.78	1.17 ± 0.41	10.17 ± 3.29	16.33 ± 3.05
P6, during ETI (W1282R/CFTRdele2,3)	−20.17 ± 6.21	24.17 ± 6.02	39.17 ± 3.56	38.33 ± 7.18
P4, during ETI (W1282R/CFTRdele2,3)	−5.83 ± 0.54	13.33 ± 2.30	39.47 ± 3.57	33.33 ± 10.38
P8, during ETI (W1282R/394delTT)	−23.50 ± 8.09	20.17 ± 0.29	25.33 ± 1.67	28.33 ± 3.78
F508del/F508del [15,16]	−18.39 ± 5.62	3.06 ± 0.89	16.55 ± 1.44	21.50 ± 5.46
Healthy control (wt/wt CFTR) [15,16]	−8.98 ± 3.42	25.78 ± 4.41	117.44 ± 4.32	101.68 ± 10.99

**Table 6:** Functional Assay Findings and Clinical Outcomes in Patients Carrying the W1282R Variant.

Patient	Genotype	Clinical Outcomes During ETI	Change in Sweat Conductivity, mmol/L NaCl eq.	ICM Findings	Intestinal Organoid Findings
P4	W1282R/CFTRdele2,3	High baseline ppFEV_1_, stable; BMI +0.5 kg/m^2^	−3	Marked CFTR-mediated chloride transport detected during ETI	FIS response to CFTR modulators
P6	W1282R/CFTRdele2,3	ppFEV_1_ +6 percentage points; BMI +2.4 kg/m^2^	−2	Marked increase in CFTR-mediated chloride transport during ETI	ND
P8	W1282R/394delTT	ppFEV_1_ ND; BMI −0.3 kg/m^2^	+1	Marked CFTR-mediated chloride transport detected during ETI	FIS response to CFTR modulators

BMI, body mass index; CFTR, cystic fibrosis transmembrane conductance regulator; ETI, elexacaftor/tezacaftor/ivacaftor; FIS, forskolin-induced swelling; ICM, intestinal current measurement; ND, not done; ppFEV_1_, percent predicted forced expiratory volume in 1 s.

## Data Availability

The data presented in this study are available from the corresponding author upon reasonable request. The data are not publicly available due to privacy and ethical restrictions related to patient confidentiality.
